# Evolution of right ventricular contraction patterns of adult heart transplant recipients by three-dimensional echocardiography

**DOI:** 10.3389/fcvm.2026.1885027

**Published:** 2026-09-11

**Authors:** Yuji Xie, Wei Sun, Linyue Zhang, Yanting Zhang, Shuangshuang Zhu, Yun Yang, Xin Zhang, Sushan Xiao, Liqiong Shi, Qing Lv, Yuman Li, Chun Wu, Li Zhang, Mingxing Xie

**Affiliations:** 1 Department of Ultrasound Medicine, Union Hospital, Tongji Medical College, Huazhong University of Science and Technology, Wuhan, Hubei, China; 2 Clinical Research Center for Medical Imaging in Hubei Province, Wuhan, Hubei, China; 3 Hubei Province Key Laboratory of Molecular Imaging, Wuhan, Hubei, China

**Keywords:** 3D echocardiography, cardiac function, heart transplantation, normal reference value, right ventricle

## Abstract

**Background:**

The right ventricular contractile pattern is altered after heart transplantation (HT). This study aims to use three-dimensional echocardiography (3DE) to comprehensively assess right ventricular contractile pattern in HT patients.

**Methods:**

In a cohort of 200 adult HT recipients, we serially assessed 3DE RV contraction patterns—including global (RVEF), longitudinal (LEF), radial (REF), and anteroposterior (AEF) ejection fractions—from the early post-operative period to 3 years. 50 HT patients were compared to matched healthy controls and open-heart surgery patients. Sex-specific reference values were derived.

**Results:**

RV function parameters improved within the first year (all *P* < 0.05), then stabilized. At 1 year, HT recipients exhibited a distinct 3D contraction pattern compared to controls: attenuated longitudinal and anteroposterior function (LEF: 18.2% vs. 28.6%; AEF: 23.8% vs. 25.4%; *P* < 0.05) with compensatory enhanced radial contraction (REF: 29.8% vs. 24.6%; *P* < 0.05). Established reference ranges confirmed this phenotype. Cardiopulmonary bypass time were key determinants of this pattern.

**Conclusions:**

HT recipients develop a unique, stabilized RV contraction pattern by one year, characterized by a shift from longitudinal to radial dominance.

## Background

Heart transplantation (HT) remains the definitive therapy for end-stage heart failure. While survival has improved, right ventricular (RV) dysfunction post-HT is a major determinant of graft failure and mortality (1–9). Accurate assessment of RV function is therefore critical for post-transplant management.

The RV has a complex contraction pattern, involving longitudinal, radial, and anteroposterior components. In health, longitudinal contraction predominates (10). This pattern is altered after cardiac surgery, with a shift toward radial dominance, a change most pronounced following HT (11–13). Conventional two-dimensional echocardiography (2DE) is limited in capturing this three-dimensional (3D) remodeling, particularly in the transplanted heart where geometric assumptions fail.

Novel three-dimensional echocardiography (3DE) techniques have overcome these limitations, allowing direct quantification of global and directional RV ejection fractions (14–17) and show promises for predicting adverse cardiovascular events (18–21). Lakatos et al. developed commercial software that facilitates accurate assessment of the three-dimensional RV contractile pattern (22). While initial studies describe a single post-HT timepoint (23), RV function is known to evolve dynamically after surgery (24–27). Crucially, the trajectory of this 3D adaptation, its time to stabilization, and—most importantly—its clinical relevance for patient management remains unknown.

Therefore, we aimed to (1) characterize the evolution of the 3D RV contraction pattern after HT until stabilization; (2) identify clinical and imaging determinants of this pattern; (3) compare the stabilized pattern in HT recipients to healthy controls and other surgical patients; and (4) establish sex-specific reference values for this population. Ultimately, we seek to define a novel 3D echocardiographic phenotype that may aid in the early detection of RV dysfunction post-transplantation.

## Methods

### Study population

This was a retrospective analysis of prospectively enrolled cohort who underwent orthotopic HT at Union Hospital, Tongji Medical Center between January 1st 2018 and December 30th 2023. The study included 200 clinically stable adult (≥18 years) HT recipients. Clinical stability at the time of echocardiography was defined as: no acute cellular or antibody-mediated rejection (biopsy-proven), no cardiac allograft vasculopathy (angiography/CTA), no pulmonary hypertension (mean PAP ≤ 20 mmHg), no significant arrhythmia or valvular disease, and preserved biventricular function (LVEF > 50%, RVFAC > 35%). From an initial 250 patients, 50 were excluded for missing (*n* = 15), unanalyzable 3D images (*n* = 30; poor acoustic window/frame rate ≤ 18 Hz), and multi-organ transplantation (*n* = 5).

All patients had a complete echocardiogram in the early postoperative period (<3 months) and at least one follow-up study at 6 months, 1, 2, or 3 years. Early postoperative TTE was defined as the first comprehensive research-quality echocardiogram performed in the outpatient clinic after hospital discharge. In our cohort, this was obtained at a median of 50 days (IQR: 43–59) post-transplantation.

For comparison, 50 age- and sex-matched healthy controls were enrolled from routine health check-ups. In addition, 50 patients who underwent open-heart surgery (20 after coronary artery bypass grafting, 15 after aortic valve replacement, 15 after mitral valve replacement) without postoperative complications were retrospectively selected from the echo database during the same period. For patients after open heart surgery, the inclusion criteria are as follows: normal cardiac function (LVEF > 50%, RVFAC > 35%), time after valve replacement or coronary artery bypass grafting ≥ 1 month, no pulmonary hypertension nor prosthetic valve complications. From the overall cohort, 50 post-transplant patients were selected to match the non-transplant cohort (*n* = 50) and healthy controls (*n* = 50) by age and sex.

To generate normative reference for RV function at time of stabilization, 500 HT patients from database were selected and analyzed.

This study is approved by the Institutional Review Board of Union Hospital, Tongji Medical College, Huazhong University of Science and Technology (UHCT21390) and complied with the Declaration of Helsinki.

### Data acquisition

#### 1 Clinical data acquisition

Clinical data of HT recipients were extracted from electronic medical records, encompassing demographics, preoperative indices (including pulmonary pressures and heart failure etiology etc.), intraoperative indices (cardiopulmonary bypass time etc.), and postoperative indices (complications, medications etc.).

#### 2 2DE and 3DE image acquisition

##### Two-dimensional image acquisition

Examinations were performed on a Philips EPIC 7C system (S5-1, S8-3 transducers) with simultaneous ECG tracing. Standard views for RV assessment (including RV inflow, outflow, focused apical 4-chamber, and parasternal views) were optimized for endocardial definition. For each view, cine loops of five cardiac cycles were stored (frame rate: 45–70 Hz).

##### Three-dimensional image acquisition

Full-volume RV datasets were acquired from the apical window using an X5-1 matrix transducer. Sector dimensions were adjusted to encompass the entire RV. For each acquisition, cine loops of five cardiac cycles were stored (frame rate: 18–23 Hz) in multi-beat technique using the Heart Model automated quantification module. All images were archived in DICOM format.

#### 3 2DE parameter quantification

Measurements followed ASE guidelines (18). From the RV-focused apical 4-chamber view, basal (RVD1), mid (RVD2), and longitudinal (RVD3) dimensions were obtained. Tricuspid annular plane systolic excursion (TAPSE) was measured by M-mode, and the systolic velocity (S’) by tissue Doppler imaging (TDI).

The RV end-diastolic and end-systolic areas (RVEDA, RVESA) were planimetered to calculate the fractional area change (RVFAC). From the TDI spectral trace at the lateral tricuspid annulus, isovolumic times (IVCT, IVRT) and ejection time (ET) were measured to calculate the RV myocardial performance index (MPI).

#### 4 3DE data post-processing

3D RV datasets were analyzed offline using QLAB (v13.0) with the 3D Auto RV module. After loading the dataset, key anatomical landmarks (Ventricular apex, midpoints of mitral and tricuspid annuli) were manually identified.

The software performed semi-automated endocardial border detection. The border was then manually adjusted slice-by-slice in both short- and long-axis views to ensure accurate tracking, excluding trabeculations from the myocardial mass. Upon finalization, the software generated a dynamic RV model and calculated end-diastolic volume, end-systolic volume, and RVEF.

#### 5 3D parameter quantification

A software named ReVISION (Right Ventricular Separate wall motIon quantificatiON), developed by Lakatos et al. using the Unity3D engine, was employed to quantify RV volumetric changes along different directional components (22). This method first decomposes the exported RV Beutel mesh model along three axes, ensuring alignment of the orthogonal axes with the anatomical directions of the RV (longitudinal, radial, and anteroposterior), then calculates the corresponding RV volumetric components for each axis based on mesh vertex displacement.

The dynamic 3D RV model was exported from QLAB and imported into ReVISION software (Argus Cognitive) for directional motion analysis (Figure 1). The software automatically adjusted the model orientation and decomposed global RV motion into longitudinal, anteroposterior, and radial components using a mesh vertex method (26). This yielded the following parameters: longitudinal ejection fraction (LEF), radial ejection fraction (REF), anteroposterior ejection fraction (AEF), their ratios to RVEF (LEF/RVEF, REF/RVEF, AEF/RVEF), and 3D RV global longitudinal strain (RVGLS_3d_).

**Figure 1:**
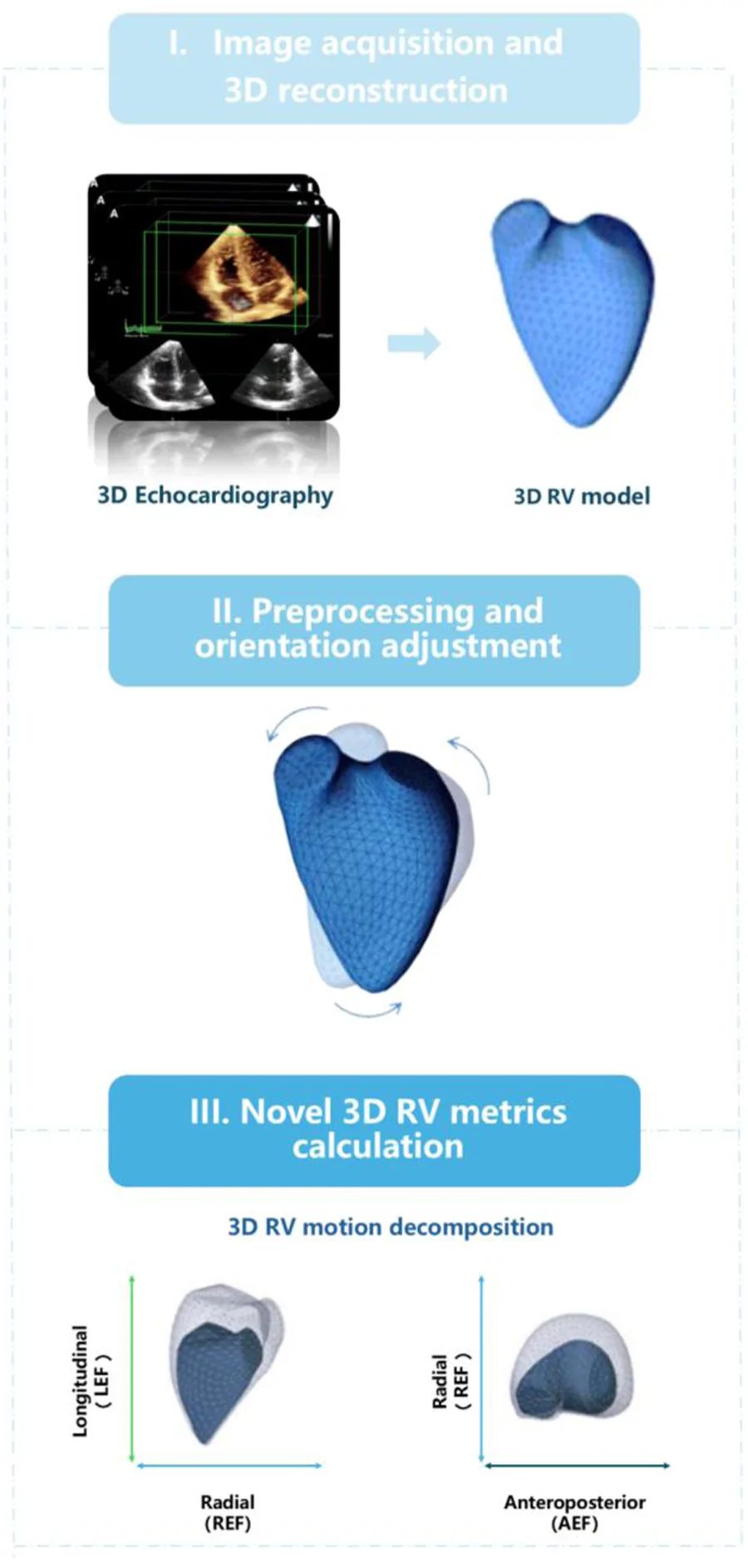
Workflow schematic illustrating the decomposition of right ventricular (RV) contraction into its three principal component. (I) Image Acquisition and 3D Right Ventricular Model Reconstruction; (II) Preprocessing of Motion Reconstruction Model and Orientation Standardization Adjustment; (III) Acquisition of 3D Right Ventricular Systolic Pattern Parameters: LEF, REF, and AEF.

### Statistical analysis

Data normality was assessed using the Shapiro–Wilk test. Continuous variables are presented as mean ± standard deviation or median (interquartile range), as appropriate. Categorical data are presented as frequencies (percentages).

Longitudinal changes in echocardiographic parameters were analyzed using a linear mixed-effects model with Geisser-Greenhouse correction and *post-hoc* Welch's tests, treating time as a categorical factor. All reported *P*-values for between-timepoint comparisons are derived from these *post-hoc* tests. Cross-sectional comparisons between groups were performed using ANOVA with Bonferroni *post-hoc* tests or Welch's *t*-test. Categorical variables were compared using the Chi-square or Fisher's exact test. Sensitivity analysis excluding to test the robustness of our findings.

Pearson correlation was used to assess relationships between clinical/imaging variables and RV contraction parameters. Normal reference ranges were defined using the 2.5th and 97.5th percentiles.

Intra- and inter-observer reproducibility for 3D RV parameters (assessed in 20 random patients by the same observer at a one-month interval and by an independent observer, respectively) was evaluated using intraclass correlation coefficients (ICCs).

## Results

### Clinical characteristics

A total of 200 adult HT recipients [80.5% male, median age 48 (IQR: 38–56)] were enrolled. The baseline demographic and clinical characteristics of the study population are presented in Table 1. Pre-transplant characteristics included severely reduced biventricular function (LVEF: 26.0 ± 9.8%, RV FAC: 24.6 ± 8.6%) and prevalent pulmonary hypertension (63.0%, median mPAP: 29.0 mmHg). The leading etiology was dilated cardiomyopathy (61.0%).

**Table 1:** Preoperative, perioperative, and postoperative clinical data of HT patients (*N* = 200).

Variable	Value
Recipient gender [*n*(%)]	
Male	161 (80.5)
Female	39 (19.5)
Age at transplantation [years]	48 (38, 56)
Preoperative	
New York Heart association functional class [*n*(%)]	
III	12 (6.0)
IV	188 (94.0)
6 Minute Walk Distance [m]	220 (100, 245)
Left ventricular ejection fraction [%]	26.0 (19.4, 32.6)
Right ventricular fractional area change [%]	24.6 (18.8, 30.4)
Pulmonary artery systolic pressure [mmHg]	43.0 (32.2, 53.8)
Mean pulmonary artery pressure [mmHg]	29.0 (19.0, 37.0)
Pulmonary hypertension [*n*(%)]	126 (63)
Right atrial pressure [mmHg]	8.0 (6.0, 10.0)
Pulmonary artery wedge pressure [mmHg]	17.5 (10.0, 23.0)
Total pulmonary resistance [dynes·s·cm^−5^]	540 (267, 770)
Pulmonary vascular resistance [dynes·s·cm^−5^]	247 (177, 316)
Transpulmonary gradient [mmHg]	11.0 (9.0, 15.0)
Etiology of transplantation [*n*(%)]	
Dilated cardiomyopathy	122 (61.0)
Hypertrophic cardiomyopathy	2 (1.0)
Ischemic cardiomyopathy	45 (22.5)
Valvular cardiomyopathy	22 (11.0)
Others	9 (4.5)
Preoperative comorbidities [*n*(%)]	
Hypertension	58 (29.0)
Hyperlipidemia	105 (52.5)
Hyperglycemia	89 (44.5)
Cerebrovascular disease	20 (10.0)
Renal dysfunction	52 (26.0)
Transplantation technique [*n* (%)]	
Bicaval	172 (86.0)
Biatrial	28 (14.0)
Ascending aorta clamp time [min]	32 (24, 40)
Cardiopulmonary bypass time [min]	118 (96, 140)
Ischemic time [min]	85 (64.8, 105.3)
Mechanical circulatory support usage [*n*(%)]	
IABP	48 (24.0)
ECMO	4 (2.0)
ICU stay [days]	11 (8, 16)
Postoperative hospital stay [days]	45 (35, 53)
Postoperative	
Postoperative comorbidities [*n*(%)]	
Hypertension	62 (31.0)
Hyperlipidemia	105 (52.5)
Hyperglycemia	95 (47.5)
Cerebrovascular disease	20 (10.0)
Renal dysfunction	52 (26.0)
Postoperative medications [*n*(%)]	
ACEI	124 (62.0)
ARB	175 (87.5)
*β*-Blocker	73 (36.5)
Calcium channel blocker	198 (99.0)
Tacrolimus	200 (100.0)
Corticosteroids	185 (92.5)
Mycophenolate mofetil	124 (62.0)
Cyclosporine	175 (87.5)

Continuous variables are presented median (interquartile range), and categorical variables as count (percentage). ACEI, angiotensin-converting enzyme inhibitor; ARB, angiotensin II receptor blocker; IABP, intra-aortic balloon pump; ECMO, extracorporeal membrane oxygenation; HT, heart transplantation.

Common pre-operative comorbidities were dyslipidemia (52.5%) and hyperglycemia/diabetes (44.5%). Surgery employed bicaval technique in 86.0% of patients, with a mean cardiopulmonary bypass time of 118 ± 32 min. Peri-operative mechanical support was used in 24.5% of patients (IABP: 24.0%, VA-ECMO: 2.0%). The median ICU stay was 11 days.

Dyslipidemia (52.5%) and hyperglycemia/diabetes (47.5%) remained most prevalent post-transplant complication. Immunosuppression was based on tacrolimus (100.0%), with most patients receiving corticosteroids (92.5%) and cyclosporine (87.5%). Concomitant medications included calcium channel blockers (99.0%) and renin–angiotensin system inhibitors (ACEi/ARBs, 87.5–62.0%).

During the 3-year observational period, post-transplant AF occurred in 3% (6 out of 200 patients) of the overall cohort. Most episodes were transient, with only 1.0% (2 out of 200 patients) requiring medical management.

### Evolution of RV contraction pattern

A total of 200 patients were enrolled at baseline, of whom 131 completed the 1-year and 78 the 3-year follow-up. The primary reasons for non-attendance were COVID-19-related travel restrictions and non-local residence. Baseline characteristics of 3-year completers (*n* = 78) vs. non-completers (*n* = 122) are summarized in Supplementary Table 6. Apart from transplantation etiology, no significant differences were observed between the two groups (all *P* > 0.05).

Echo examinations were completed at the following intervals: early post-operative (median 50 days, IQR: 43–59; *n* = 200), 6 months (median 210 days, IQR: 150–210; *n* = 178), 1 year (median 365 days, IQR: 300–420; *n* = 131), 2 years (median 730 days, IQR: 630–810; *n* = 100), and 3 years (median 1,095 days, IQR: 960–1,200; *n* = 78).

The RV contraction pattern evolved dynamically, with initially diminished global and directional functions that improved significantly throughout the first year and stabilized thereafter (Table 2, Figure 2). Diastolic and volumetric data are provided in Supplementary Tables 1, 2.

**Table 2:** Changes in three-dimensional right ventricular contraction patterns after HT.

	Postoperative time point					ANOVA *P-*value
	Postoperative Early (*N* = 200)	6 months (*N* = 178)	1 year (*N* = 131)	2 years (*N* = 100)	3 years (*N* = 78)	
Contraction pattern parameters						
Global function						
RVEF [%]	44.6 (42.5, 47.0)	50.1 (49.2, 51.3)*	53.4 (50.6, 56.9)*,^#^	53.5 (50.5, 56.6)*,^#^	53.5 (49.9, 56.5)*,^#^	**<0** **.** **0001**
Longitudinal contraction function						
LEF [%]	13.0 (9.8, 17.1)	16.4 (14.5, 18.8)*	18.2 (14.5, 21.8)*,^#^	18.3 (14.5, 22.2)*,^#^	18.4 (14.7, 22.4)*,^#^	**<0** **.** **0001**
LEF/RVEF	0.29 (0.23, 0.37)	0.32 (0.28, 0.38)*	0.34 (0.29, 0.41)*,^#^	0.34 (0.27, 0.41)*,^#^	0.34 (0.28, 0.40)*,^#^	**<0** **.** **0001**
Radial contraction						
REF [%]	22.0 (18.1, 25.7)	26.5 (23.9, 29.7)*	29.8 (25.3, 33.1)*,^#^	29.9 (25.4, 33.2)*,^#^	29.9 (25.5, 33.0)*,^#^	**<0** **.** **0001**
REF/RVEF	0.49 (0.41, 0.58)	0.53 (0.48, 0.58)*	0.55 (0.50, 0.58)*,^#^	0.55 (0.50, 0.58)*,^#^	0.55 (0.51, 0.58)*,^#^	**0** **.** **0126**
Anteroposterior contraction						
AEF [%]	19.9 (16.9, 22.8)	22.5 (18.5, 27.2)*	23.8 (20.0, 27.3)*,^#^	23.8 (20.0, 27.3)*,^#^	23.9 (20.1, 27.4)*,^#^	**<0** **.** **0001**
AEF/RVEF	0.42 (0.38, 0.50)	0.43 (0.34, 0.53)*	0.44 (0.40, 0.48)*,^#^	0.44 (0.40, 0.48)*,^#^	0.44 (0.40, 0.48)*,^#^	**<0** **.** **0001**
Conventional and strain parameters						
RVFAC [%]	41.5 (39.5, 44.8)	43.2 (40.3, 46.1)*	45.1 (42.3, 48.7)*,^#^	45.8 (42.4, 48.9)*,^#^	45.0 (42.5, 47.9)*,^#^	**<0** **.** **0001**
RVGLS_3d_ [%]	−14.2 (−16.0, −12.0)	−16.2 (−17.9, −14.6)*	−16.5 (−18.3, −14.9)*	−16.7 (−18.7, −15.0)*	−16.9 (−19.0, −15.1)*	**<0.0001**
TAPSE [cm]	1.1 (1.0, 1.3)	1.3 (1.1, 1.6)*	1.3 (1.1, 1.5)*	1.4 (1.2, 1.6)*	1.4 (1.2, 1.6)*	**<0** **.** **0001**
S’ [cm/s]	9.1 (8.3, 10.0)	9.8 (8.4, 11.1)*	10.0 (8.5, 11.2)*	10.3 (9.2, 11.3)*	10.8 (8.6, 11.9)*	**<0** **.** **0001**

Data are presented as median (interquartile range). 3D-RVGLS, three-dimensional right ventricular global longitudinal strain; AEF, anteroposterior ejection fraction; AEF/RVEF, anteroposterior ejection fraction/right ventricular ejection fraction; LEF, longitudinal ejection fraction; LEF/RVEF, longitudinal ejection fraction/right ventricular ejection fraction; REF, radial ejection fraction; REF/RVEF, radial ejection fraction/right ventricular ejection fraction; RVEF, right ventricular ejection fraction; RVFAC, right ventricular fractional area change; RVGAS, right ventricular global area strain; RVGCS, right ventricular global circumferential strain; S′, tricuspid annular peak systolic velocity; TAPSE, tricuspid annular plane systolic excursion; TFAC, transverse fractional area change. Bold values indicate statistically significant differences between groups (*p* < 0.05).

* Statistically significant difference compared to baseline.

^#^ Statistically significant difference compared to 6 months postoperatively.

**Figure 2:**
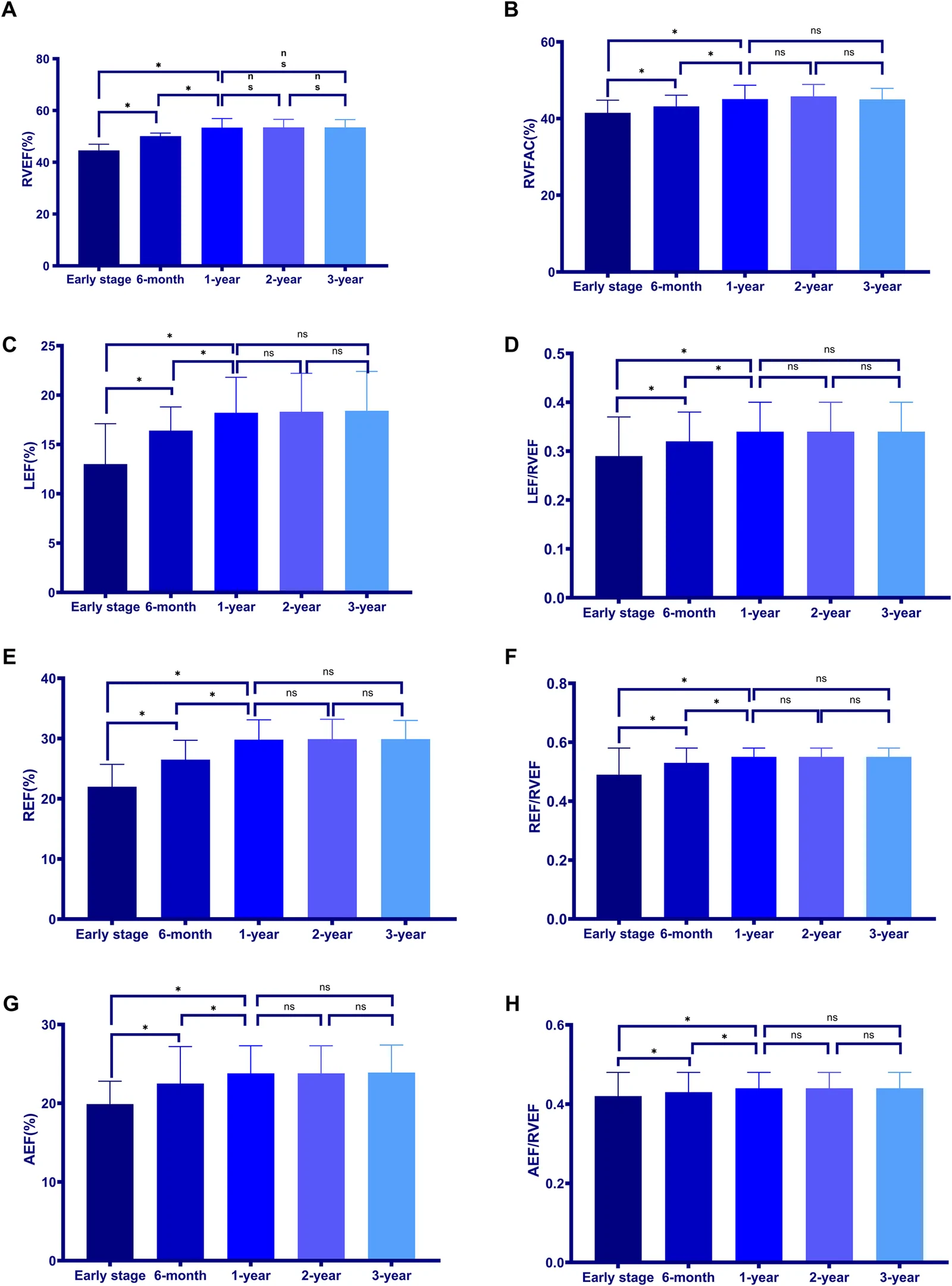
Evolution of 3D right ventricular contractile patterns after heart transplantation at early postoperative period, 6 months, 1, 2, and 3 years. **(A)** RVEF, **(B)** RVFAC, **(C)** LEF, **(D)** LEF/RVEF, **(E)** REF, **(F)** REF/RVEF, **(G)** AEF, **(H)** AEF/RVEF. AEF, anteroposterior ejection fraction; LEF, longitudinal ejection fraction; REF, radial ejection fraction; RVEF, right ventricular ejection fraction; RVFAC, right ventricular fractional area change. *P < 0.05; ns, not statistically significant.

RVEF increased from 44.6% in the early period to 50.1% at 6 months and to 53.4% at 1 year (both *P* < 0.001), with no significant change thereafter. Similarly, longitudinal function improved markedly: LEF increased from 13.0% to 16.4% to 18.2% (*P* < 0.001 at each interval), and the LEF/RVEF ratio rose from 0.29 to 0.34. REF increased from 22.0% to 26.5% to 29.8% (*P* < 0.001), with the REF/RVEF ratio increasing from 0.49 to 0.55. AEF also rose, from 19.9% to 22.5% to 23.8% (*P* < 0.001), while the AEF/RVEF ratio increased from 0.42 to 0.44. No parameter changed significantly between 1- and 3-years post-transplantation (all *P* > 0.05), confirming functional stabilization by the first year.

### Distinct RV contraction pattern in HT patients

A distinct RV contraction pattern was identified in HT recipients compared to healthy controls and non-HT open-heart surgery patients (Table 3, Supplementary Figure 2). All parameters were assessed at one-year post-operation. HT recipients exhibited a higher BMI and heart rate than both control groups.

**Table 3:** Comparison of three-dimensional right ventricular contraction patterns at 1 year post-HT with other populations.

	Transplant patients	Cardiac surgery	Healthy controls	ANOVA *P-*value
	(*N* = 50)	(*N* = 50)	(*N* = 50)	
Demographics				
Age [years]	48.0 (39.2, 56.8)	47.5 (40.1, 54.9)	47.0 (37.6, 56.5)	0.666
Gender [*n*(%)]				
Male	26 (52)	28 (56)	25 (50)	0.948
Female	24 (48)	22 (44)	25 (50)	
Height [cm]	171.9 (166.5, 177.3)	169.4 (164.7, 174.1)	172.1 (167.3, 176.9)	0.887
Weight [kg]	68.2 (64.9, 71.5)*,^#^	66.8 (63.4, 70.2)*	65.3 (61.9, 68.7)	**<0** **.** **001**
BMI [kg/m^2^]	23.6 (20.0, 27.2)*,^#^	23.4 (19.8, 27.0)*	22.1 (19.9, 24.4)	**<0** **.** **001**
HR [bpm]	88.0 (80.6, 95.5)*,^#^	84.0 (73.9, 94.2)*	70.0 (62.6, 77.5)	**<0** **.** **001**
Time from surgery to echocardiography [days]	381 (274, 491)	391 (346, 534)	/	0.282
Global function				
RVEF (%)	53.4 (52.1, 54.8)*,^#^	54.5 (53.1, 55.9)	57.2 (55.9, 58.6)	**<0** **.** **001**
Longitudinal function				
LEF (%)	18.2 (16.1, 20.4)*,^#^	20.5 (18.4, 22.6)*	28.6 (26.6, 30.7)	**<0** **.** **001**
LEF/RVEF	0.30 (0.27, 0.34)*,^#^	0.40 (0.37, 0.44)*	0.50 (0.45, 0.55)	**<0** **.** **001**
Radial function				
REF (%)	29.8 (27.6, 32.1)*,^#^	28.4 (25.9, 30.9)*	24.6 (22.6, 26.7)	**<0** **.** **001**
REF/RVEF	0.55 (0.50, 0.60)*	0.52 (0.47, 0.57)*	0.43 (0.39, 0.47)	**<0** **.** **001**
Anteroposterior function				
AEF (%)	23.8 (21.6, 26.1)*	24.5 (22.6, 26.5)	25.4 (23.4, 27.5)	**<0** **.** **001**
AEF/RVEF	0.44 (0.40, 0.48)	0.45 (0.42, 0.49)	0.44 (0.40, 0.48)	0.564

BMI, body mass index; HR, heart rate; RVEF, right ventricular ejection fraction; LEF, longitudinal ejection fraction; REF, radial ejection fraction; AEF, anteroposterior ejection fraction; RVGLS3d, three-dimensional right ventricular global longitudinal strain; 3D-RVGCS, three-dimensional right ventricular global circumferential strain; 3D-RVGAS, three-dimensional right ventricular global area strain. Bold values indicate statistically significant differences (*p* < 0.05).

* Statistically significant difference compared to healthy controls.

^#^ Statistically significant difference compared to post-thoracotomy patients.

Global RV systolic function, while within normal range, was lower in both surgical groups than in healthy controls (RVEF: 53.4 ± 2.0% and 54.5 ± 2.1% vs. 57.2 ± 2.0%, *P* < 0.001). Longitudinal function was markedly impaired in surgical patients, with the greatest reduction in the HT cohort (LEF: 18.2 ± 3.2% vs. 20.5 ± 3.1% vs. 28.6 ± 3.0%; LEF/RVEF: 0.30 ± 0.05 vs. 0.40 ± 0.05 vs. 0.50 ± 0.07; all *P* < 0.001). Conversely, radial function was increased, most prominently in HT recipients (REF: 29.8 ± 3.3% vs. 28.4 ± 3.1% vs. 24.6 ± 3.0%; REF/RVEF: 0.55 ± 0.07 vs. 0.52 ± 0.06 vs. 0.50 ± 0.06; all *P* < 0.001). Anteroposterior contraction was also significantly weaker in HT patients compared to healthy controls (AEF: 23.8 ± 3.3% vs. 24.5 ± 2.9% vs. 25.4 ± 3.0%, *P* < 0.001).

### Proportion of normalized RV function

The proportion of patients with normal RV contraction parameters increased progressively during the first post-transplant year (Figure 3). Normality was defined using ASE guidelines for RVEF and published reference values for directional components (28, 29). In the early postoperative period, longitudinal function was most frequently impaired, with only 46% of patients having a normal LEF, compared to 73% for RVEF, 83% for REF, and 84% for AEF. By 6 months, recovery was evident, with corresponding proportions increasing to 82% (RVEF), 58% (LEF), 100% (REF), and 89% (AEF). At 1-year, further normalization occurred: 88% (RVEF), 65% (LEF), 100% (REF), and 95% (AEF).

**Figure 3:**
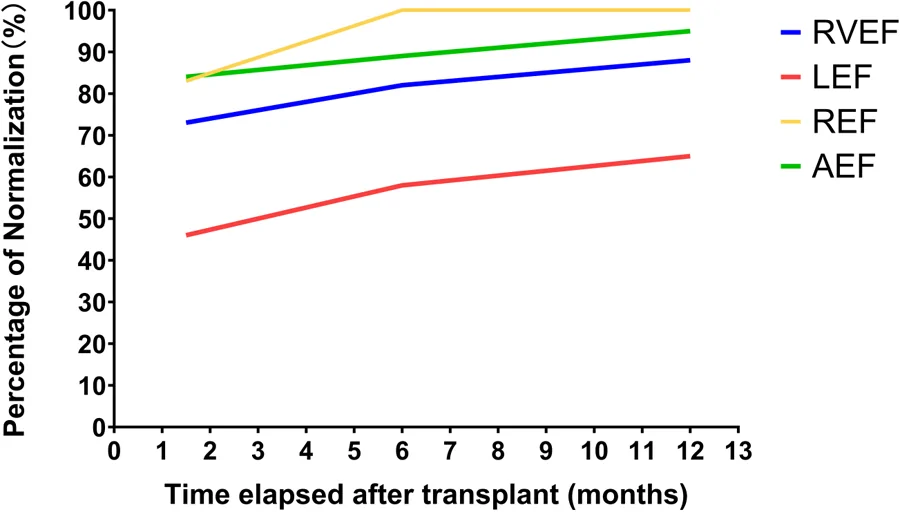
Proportion of normalized right ventricular functional parameters of adults heat transplantation patient within One year after surgery. Proportions of normalized right ventricular contraction pattern parameters at early stage, 6 months and 1 year.

### Normal reference for RV contraction pattern

After identifying 1-year post-transplant as the stabilization point from our longitudinal analysis, we extended our database search to identify all adult HT recipients who had undergone 3D echocardiography at the 1-year time point, yielding a total of 500 patients. As summarized in Table 4, the mean values at 1 year confirmed a distinct, stabilized phenotype: HT patients exhibited lower global, longitudinal, and anteroposterior function, but higher radial function, compared to healthy population normal reference (all *P* < 0.001).

**Table 4:** Comparison of three-dimensional right ventricular contraction pattern parameters at 1 year post-HT with normal reference values.

Parameter	HT patients	Normal reference values*	Welch *P-*value
	(*N* = 131)	(*N* = 2,262)	
RVEF (%)	53.4 ± 2.0	55.5 ± 5.8	**<0** **.** **05**
LEF (%)	18.2 ± 3.2	21.6 ± 5.7	**<0** **.** **05**
REF (%)	29.8 ± 3.3	29.0 ± 7.0	**<0** **.** **05**
AEF (%)	23.8 ± 3.3	25.4 ± 5.9	**<0** **.** **05**
3D-RVGLS (%)	−16.5 ± 1.8	−19.7 ± 3.7	**<0** **.** **05**

3D-RVGLS, three-dimensional right ventricular global longitudinal strain; AEF, anteroposterior ejection fraction; LEF, longitudinal ejection fraction; REF, radial ejection fraction; RVEF, right ventricular ejection fraction. Bold values indicate statistically significant differences (*p* < 0.05).

* Reference values for three-dimensional right ventricular contraction patterns in healthy populations from Cotella et al. (29).

Sex-specific reference ranges for 3D RV parameters are presented in Supplementary Table 3. female HT recipients had significantly smaller RV volumes but superior global and directional contractile function (RVEF: 53% vs. 50%; LEF: 22% vs. 17%; REF: 28% vs. 23%; AEF: 27% vs. 22%; all *P* < 0.05). Derived normal ranges were: RVEF: 39%–60% (males) vs. 41%–64% (females); LEF: 6%–28%; REF: 7%–33% (males) vs. 11%–38% (females); and AEF: 9%–32% (males) vs. 13%–34% (females). Age-stratified reference ranges are shown in Supplementary Table 4. While RV volumes did not differ by age, younger patients exhibited significantly better RV contractile function.

### Correlates of the RV contraction pattern

Potential determinants of the RV contraction pattern are summarized in Tables 5, 6. At one-year post-operation, RVEF showed weak negative correlations with pre-operative mean pulmonary artery pressure (*r* = −0.247), pulmonary vascular resistance (*r* = −0.278), and transpulmonary pressure gradient (*r* = −0.294; all *P* < 0.05). LEF correlated negatively with cardiopulmonary bypass time (*r* = −0.216; *P* < 0.05) and aortic cross-clamp time (*r* = −0.327; *P* < 0.05).

**Table 5:** Correlation analysis of adult post-HT right ventricular contraction patterns with clinical indicators.

Variable	RVEF		LEF		REF		AEF	
	*r*	*P-*value	*r*	*P-*value	*r*	*P-*value	*r*	*P-*value
Preoperative data								
Age at transplantation [years]	0.033	0.751	0.071	0.51	0.18	0.093	**−0** **.** **236**	**0** **.** **027**
6-Minute Walk Distance [m]	0.292	0.632	0.826	0.085	0.096	0.639	0.024	0.968
Left ventricular ejection fraction [%]	0.068	0.52	0.048	0.66	0.105	0.34	0.064	0.562
Right ventricular fractional area change [%]	0.118	0.429	0.095	0.542	0.176	0.258	0.169	0.278
Pulmonary artery systolic pressure [mmHg	0.117	0.106	0.038	0.739	0.107	0.356	0.015	0.894
Mean pulmonary artery pressure [mmHg]	**−0** **.** **247**	**0** **.** **024**	0.047	0.686	0.203	0.077	0.109	0.347
Right atrial pressure [mmHg]	0.009	0.447	0.025	0.836	0.033	0.789	0.059	0.634
Pulmonary artery wedge pressure [mmHg]	0.164	0.171	0.018	0.887	0.183	0.15	0.116	0.363
Total pulmonary resistance [dynes·s·cm⁻^5^]	0.173	0.138	0.014	0.907	0.168	0.17	0.167	0.171
Pulmonary vascular resistance [dynes·s·cm⁻⁵]	**−0** **.** **278**	**0** **.** **025**	0.043	0.748	0.218	0.099	0.091	0.492
Transpulmonary gradient [mmHg]	**−0** **.** **294**	**0** **.** **029**	0.032	0.824	0.102	0.482	0.122	0.401
Perioperative data								
Ascending aorta clamp time [min]	0.055	0.591	0.081	0.454	0.121	0.264	0.115	0.289
Cardiopulmonary bypass time [min]	0.098	0.341	**−0** **.** **216**	**0** **.** **044**	0.112	0.302	0.115	0.289
Ischemic time [min]	0.103	0.315	**−0** **.** **327**	**0** **.** **002**	0.188	0.079	0.09	0.402
ICU stay [days]	0.115	0.27	0.048	0.664	0.011	0.919	0.02	0.85
Postoperative hospital stay [days]	0.096	0.355	0.062	0.573	0.016	0.88	0.049	0.653
Postoperative data								
At the time of echocardiography								
BNP [pg/mL]	0.342	0.451	0.148	0.778	0.714	0.11	0.706	0.116
NT-proBNP [pg/mL]	0.188	0.879	0.963	0.173	0.742	0.467	0.322	0.79
hsTnI [ng/L]	0.729	0.1	0.814	0.093	0.887	0.044	0.827	0.083
Blood urea nitrogen [mg/dL]	0.014	0.972	0.045	0.923	0.404	0.368	0.454	0.305
Creatinine [mg/dL]	0.057	0.892	0.447	0.314	0.299	0.513	0.173	0.711

AEF, anteroposterior ejection fraction; BNP, brain natriuretic peptide; hsTnI, high-sensitivity troponin I; LEF, longitudinal ejection fraction; NT-proBNP, N-terminal pro-brain natriuretic peptide; REF, radial ejection fraction; RVEF, right ventricular ejection fraction. Bold values indicate statistically significant differences (*p* < 0.05).

**Table 6:** Correlation of adult post-HT right ventricular contraction patterns with imaging indicators.

Variable	RVEF		LEF		REF		AEF	
	*r*	*P-*value	*r*	*P-*value	*r*	*P-*value	*r*	*P-*value
Right heart structural parameters								
RVD1 [mm]	0.129	0.066	**−0.392**	**<0.001**	0.097	0.129	**−0.357**	**<0.001**
RVD2 [mm]	0.038	0.293	−0.125	0.067	−0.048	0.285	0.009	0.454
RVD3 [mm]	−0.058	0.204	−0.108	0.091	−0.03	0.362	0.001	0.494
RVEDV [mL]	−0.049	0.242	0.014	0.428	−0.023	0.393	−0.027	0.375
RVESV [mL]	0.095	0.12	0.181	0.111	−0.013	0.438	0.067	0.215
Left heart structural parameters								
LVEDV [mL]	0.065	0.132	0.254	0.806	0.522	0.595	−0.054	0.75
LVESV [mL]	0.019	0.589	0.365	0.172	0.516	0.634	0.134	0.43
LVSI	−0.029	0.412	0.051	0.239	0.526	0.201	−0.108	0.526
Left heart functional parameters								
LVEF [%]	**0.305**	**0.035**	0.089	0.133	0.098	0.125	−0.044	0.304
LVGLS_2d_ [%]	−0.196	0.273	−0.37	0.512	−0.091	0.643	−0.081	0.173
LVGLS_3d_ [%]	−0.105	0.521	−0.382	0.528	−0.142	0.751	0.357	0.881

LVGLS_2D_, two-dimensional left ventricular free wall global longitudinal strain; LVGLS_3d_, three-dimensional left ventricular free wall global longitudinal strain; AEF, anteroposterior ejection fraction; LEF, longitudinal ejection fraction; LVEDV, left ventricular end-diastolic volume; LVESV, left ventricular end-systolic volume; LVSI, left ventricular sphericity index; REF, radial ejection fraction; RVD1, right ventricular basal diameter; RVD2, right ventricular mid-cavity diameter; RVD3, right ventricular longitudinal diameter; RVEDA, right ventricular end-diastolic area; RVEDV, right ventricular end-diastolic volume; RVEF, right ventricular ejection fraction; RVESA, right ventricular end-systolic area; RVESV, right ventricular end-systolic volume; RVFAC, right ventricular fractional area change; RVSI, right ventricular sphericity index. Bold values indicate statistically significant differences (*p* < 0.05).

Among imaging factors, RVEF correlated positively with LVEF (*r* = 0.305, *P* < 0.05). In contrast, LEF and AEF correlated negatively with post-operative RV basal diameter (RVD1; *r* = −0.392 and *r* = −0.236, respectively; *P* < 0.05). In summary, pre-operative pulmonary pressure, surgical times, post-operative RV basal diameter, and LV systolic function were identified as potential determinants of the RV contraction pattern. All correlation analyses were exploratory and were not adjusted for multiple comparisons.

### Reproducibility analysis

Supplementary Table 5 summarizes the intra- and inter-observer reproducibility of the 3D RV contraction pattern parameters. All parameters demonstrated excellent reproducibility, with both intra-observer and inter-observer intraclass correlation coefficients exceeding 0.90.

## Discussion

The principal findings of this study were as follows: (1) The 3D RV contraction pattern in HT recipients demonstrated a dynamic evolution. Specifically, global, longitudinal, radial, and anteroposterior RV contractile functions were reduced in the early postoperative phase but showed progressive improvement within the first year, stabilizing at first postoperative year. (2) Compared to healthy controls and non-HT open-heart surgery patients, HT patients exhibited distinct 3D RV contraction pattern, characterized by weaker longitudinal and anteroposterior contraction but an enhanced radial contraction. (3) Normal reference of 3D RV contraction pattern parameters for HT patients varied by sex and age group. (4) Clinical determinants of the 3D RV contraction pattern included preoperative pulmonary artery pressure and cardiopulmonary bypass time. Imagological determinants included RV basal diameter and left ventricular global systolic function. (5) RV contraction pattern parameters associated with adverse outcomes.

### Principle of 3DE for evaluating RV contraction patterns

Cardiac magnetic resonance, positron emission tomography, and computed tomography are commonly used imaging techniques for cardiac assessment. However, their application is limited by contraindications (30). Echocardiography is extensively used in clinical practice for quantification of ventricular structure and function. In recent years, novel 3DE technique has been used for assessing ventricular volume and function. Studies have demonstrated that 3DE offers good reproducibility and accuracy (10, 31, 32). By semi-automated endocardial border tracking, real-time 3DE technology quickly reconstructs dynamic 3D volume, thereby obtaining global RV volumes and ejection fraction. 3DE has been employed for RV function evaluation in various diseases, including HT (23), pulmonary hypertension (33), atrial septal defect (34), and tetralogy of Fallot (35). ReVISION is the first dedicated software designed to post-process RV motion along different directional components (22). By reconstructing 3D RV motion and decomposing it into directional components, the ReVISION method enables accurate and rapid assessment of RV contraction pattern. Our study innovatively applies the ReVISION method to evaluate the evolution of RV contraction pattern in HT recipients.

### Underlying mechanisms of dynamic changes of RV contraction pattern

Our research team previously enrolled 58 adult HT patients and utilized 3DE to assess the trajectory of RV functional changes within the first year after transplantation (27). The results indicated significant increases in RV functional parameters within the first 1–6 months post-operation. These findings suggested a dynamic evolution of RV function after HT. However, previous study did not evaluate RV motion along different directional components, was limited to a 1-year follow-up period, and had a relatively small sample size. The present study expands the HT patient cohort and conducts longer-term follow-up. Our observations in 200 HT patients over a 3-year period demonstrate dynamic changes of RV contraction pattern post-HT, which stabilizes by 1-year. At the 1-year mark, HT patients exhibit weakened longitudinal and anteroposterior motion, with compensatory enhancement of radial motion.

The findings of this investigation are also consistent with prior work by Lakatos (23), Goland (24), and Palomero (27). Lakatos et al. enrolled 51 clinically stable HT patients and used the ReVISION method to characterize the 3D RV contraction pattern (23). Their results showed reduced longitudinal contraction and enhanced radial contraction in HT patients, while global RV systolic function was preserved. The study also revealed a positive correlation between longitudinal contraction indices and time within the first year, indicating dynamic changes. However, their study did not identify the stabilization point for RV function. The mechanism underlying the dynamic evolution of the RV contraction pattern is likely to be multifactorial, involving contributors such as ischemia-reperfusion injury, autonomic neural remodeling, hemodynamic alterations, immune rejection, medication effects, RV remodeling, and comorbidities/infections.

### Potential factors influencing RV contraction pattern

As mentioned, longitudinal shortening is the predominant RV motion in healthy individuals. Previous literature indicates that RV contraction patterns differ significantly in populations with congenital heart disease, cardiomyopathy, heart failure, and cardiac surgery (13). Pulmonary hypertension, tetralogy of Fallot, right heart failure, and severe left ventricular systolic dysfunction can lead to increased RV afterload, altered RV geometry, and RV myocardial interstitial fibrosis, consequently modifying the RV contraction pattern (36–39). Pulmonary hypertension, if uncontrolled long-term, imposes excessive load on the RV, thereby affecting its contractile efficiency and pattern. Rako et al. prospectively enrolled 53 pulmonary hypertension patients undergoing 3DE and categorized them based on the RV-pulmonary arterial coupling parameter Ees/Ea into no/mild, moderate, and severe RV-PA uncoupling groups (38). They found that early RV-PA uncoupling was associated with reduced longitudinal contraction, whereas severe uncoupling was linked to impaired anteroposterior contraction. Surkova et al. investigated the RV contraction pattern in 292 patients with varying degrees of left ventricular systolic dysfunction (39). They reported that in patients with mild to moderate LV dysfunction, RV longitudinal and anteroposterior contractions were significantly reduced, radial contraction was significantly increased, yet global systolic function was preserved. In contrast, patients with severe LV dysfunction exhibited significantly reduced global, longitudinal, radial, and anteroposterior RV contractions.

This study compared the RV contraction pattern between healthy controls, HT recipients, and non-HT cardiac surgery patients using 3DE. The results demonstrated that compared to healthy individuals, the surgical group exhibited a distinctly different RV contraction pattern, characterized by significantly reduced longitudinal and anteroposterior contraction, alongside significantly increased radial contraction. Furthermore, the alteration in the RV contraction pattern was more pronounced in HT recipients compared to other cardiac surgery patients. This suggests that cardiac surgery itself is a significant factor inducing changes in the RV contraction pattern. Raina et al. retrospectively analyzed the RV contraction pattern in 54 HT patients, 23 CABG patients, and 25 lung transplantation patients (11). Additionally, Keyl, Dalén, Lindqvist, and other researchers have reported significant alterations in the RV contraction pattern following aortic valve replacement surgery (40–42). These researchers attributed the reduced longitudinal contraction to pericardiotomy. With the pericardium open, cardiac motion is less constrained, potentially allowing for greater transverse movement within the mediastinum and reduced longitudinal excursion. The extent of pericardial resection may directly influence the degree of change in the RV contraction pattern. Patients undergoing full sternotomy for valve surgery exhibit more pronounced reduction in RV longitudinal contraction compared to those undergoing minimally invasive valve surgery (42, 43).

The negative correlations between CPB/ischemic time and RV longitudinal function suggest that surgical insult is the determinant correlate of RV contraction pattern rather than other indices; their association with postoperative function may be mediated by their influence on surgical tolerance. The weak correlations observed in Table 5 and Table 6 should be interpreted as exploratory and hypothesis-generating, rather than as strong predictive relationships.

### Normal reference values for 3D RV in HT population

The RV contraction pattern in HT recipients is distinctly different from that in healthy individuals, indicating that normal reference values derived from healthy populations are not applicable to the post-HT population. Applying cut-off values for RV longitudinal function indices from healthy subjects (e.g., TAPSE ≥ 16 mm or S′ ≥ 9.5 cm/s) to assess the HT patients' RV function could lead to misinterpretation.

While previous studies have established normal reference values for 3D RV contraction pattern parameters in large healthy cohorts (29, 44), no study has yet investigated normal reference ranges for clinically stable HT recipients. This study is the first to analyze normal reference values for 3D RV contraction pattern parameters in HT patients, stratified by sex and age. The results indicate that the mean values for RVEF, LEF and AEF in clinically stable HT patients are lower than those in healthy populations, whereas the mean value for REF is higher.

As for predictive value, this study preliminarily suggested that an association between RV contraction pattern parameters and adverse outcomes. This is consistent with previous studies. Kitano et al. found that 3D RV contraction pattern parameters were associated with cardiac death (45). Tolvaj et al. reported that reduced RV circumferential strain was associated with all-cause mortality (46). Establishing normal reference values for 3D RV contraction pattern in the HT population holds significant potential for the early detection of RV dysfunction, optimization of treatment strategies, and improvement of patient outcomes.

## Limitation

Firstly, this was a single-center study, and the analysis was limited to the changes in the RV contraction pattern within three years after HT. Future studies should aim to expand the sample size and incorporate multi-center data to investigate the long-term evolution of right ventricular morphology and function in the HT population. Secondly, the cross-sectional design of this study, while utilizing Pearson correlation analysis to reveal associations between variables, precludes the establishment of causal relationships. Subsequent studies are planned to employ a prospective design and utilize logistic regression analysis to identify clinical and imaging factors with predictive value for the RV contraction pattern. Thirdly, the exclusion of patients with unanalyzable 3DE RV images may have introduced selection bias. Fourthly, while the inclusion of patients with preserved baseline cardiac function enhanced the homogeneity of study cohort, it also constitutes a limitation, as our results may not be directly applicable to patients with impaired baseline function. Lastly, the 3D RV parameters in this study were acquired using Philips QLAB 13.0 software. It is important to note that potential discrepancie may exist among different vendors’ post-processing analysis software (e.g., TomTec, EchoPAC).

## Conclusion

This study establishes that the 3D RV contraction pattern in HT recipients evolves dynamically, stabilizing one-year post-operation. It is distinct from healthy controls and other surgical patients, characterized by preserved global function but a shifted contribution from directional components: weakened longitudinal/anteroposterior and enhanced radial contraction. We provide specific reference values for this unique HT phenotype, which are crucial for accurate assessment and early detection of RV dysfunction in this population.

## Data Availability

The data that support the findings of this study are available from the corresponding author upon reasonable request. Requests to access these datasets should be directed to xiemx@hust.edu.cn.
