# Correction: Evolution of right ventricular contraction patterns of adult heart transplant recipients by three-dimensional echocardiography

**DOI:** 10.3389/fcvm.2026.1995484

**Published:** 2026-09-22

**Authors:** Yuji Xie, Wei Sun, Linyue Zhang, Yanting Zhang, Shuangshuang Zhu, Yun Yang, Xin Zhang, Sushan Xiao, Liqiong Shi, Qing Lv, Yuman Li, Chun Wu, Li Zhang, Mingxing Xie

**Affiliations:** 1 Department of Ultrasound Medicine, Union Hospital, Tongji Medical College, Huazhong University of Science and Technology, Wuhan, Hubei, China; 2 Clinical Research Center for Medical Imaging in Hubei Province, Wuhan, Hubei, China; 3 Hubei Province Key Laboratory of Molecular Imaging, Wuhan, Hubei, China

**Keywords:** 3D echocardiography, cardiac function, heart transplantation, normal reference value, right ventricle

In the published article, the funding statement was incorrectly given as:

The author(s) declared that financial support was received for this work and/or its publication. This work was supported by the National Natural Science Foundation of China (Grant No. 82603865) and the Interdisciplinary Research Program of HUST (Grant No. 2026JCYJ037).

The correct funding statement is:

The author(s) declared that financial support was received for this work and/or its publication. This work is supported by National Natural Science Foundation of China (grant no. 82371990 & 82302229 & 82402304 & 82427805), the Natural Science Foundation of Hubei Province (grant no. 2024AFB071 and 2026AFB268), and the Interdisciplinary Research Program ofHUST (Grant No. 2026JCYJ037).

The original version of this article has been updated.

