# Supplementary material for: Evolution of right ventricular contraction patterns of adult heart transplant recipients by three-dimensional echocardiography
Source: Front Cardiovasc Med. 2026 Sep 11;13:1885027. doi: 10.3389/fcvm.2026.1885027 (PMC13640019; doi:10.3389/fcvm.2026.1885027)
Supplement: Supplementary file 1 [file Datasheet1.docx]

| **Supplementary Table S1. Evolution of Right Ventricular Size and Volume at Different Timepoints After HT** | | | | | | | |
| --- | --- | --- | --- | --- | --- | --- | --- |
|  | **Post-operative Timepoints** | | | | |  | ***P* value** |
|  | **Postoperative**  **(n=200)** | **6 Months**  **(n=178)** | **1 Year**  **(n=131)** | **2 Years**  **(n=100)** | **3 Years**  **(n=78)** |  |  |
| RVD1 [mm] | 37.0 (34.1, 38.9) | 35.4 (32.4, 38.8)^*^ | 34.9 (32.1, 39.0)^*^ | 35.1 (32.7, 37.1)^*^ | 35.8 (33.1, 38.5)^*^ |  | **0.0034** |
| RVD2[mm] | 31.8 (28.5, 33.6) | 27.8 (25.3, 31.2)^*^ | 27.7 (24.7, 31.8)^*^ | 28.3 (26.1, 30.5)^*^ | 28.8 (25.2, 32.0)^*#^ |  | **<0.0001** |
| RVD3 [mm] | 68.0 (64.7, 72.5) | 66.1 (61.0, 71.2)^*^ | 64.7 (60.5, 69.0)^*#^ | 64.0 (59.8, 67.3)^*#^ | 63.4 (59.8, 68.0) ^*#†^ |  | **<0.0001** |
| RVEDA [cm^2^] | 19.7 (17.2, 20.8) | 17.7 (15.1, 20.1)^*^ | 16.4 (14.2, 19.0)^*#^ | 16.8 (14.5, 18.7)^*#^ | 16.4 (14.3, 19.0)^*#^ |  | **<0.0001** |
| RVESA [cm^2^] | 11.2 (9.8, 12.3) | 10.0 (8.5, 11.5)^*^ | 8.8 (7.9, 10.5)^*#^ | 8.3 (6.9, 9.4)^*#†^ | 8.7 (7.8, 10.6)^*#‡^ |  | **<0.0001** |
| RVEDV [mL] | 87.9 (74.1, 104.2) | 85.6 (71.0, 98.2) ^*^ | 85.3 (67.8, 102.1) ^*^ | 84.8 (65.8, 106.3) | 85.5 (77.8, 97.7) |  | **0.007** |
| RVESV [mL] | 44.8 (37.6, 53.2) | 42.5 (35.5, 48.7)^*^ | 41.1 (33.2, 28.6)^*^ | 38.1 (29.0, 46.7)^*#^ | 36.1 (30.0, 44.0)^*#^ |  | **<0.0001** |
| Note: Data are presented as median (interquartile range). RVD1, right ventricular basal transverse diameter; RVD2, right ventricular mid-cavity transverse diameter; RVD3, right ventricular longitudinal diameter; RVEDA, right ventricular end-diastolic area; RVESA, right ventricular end-systolic area; RVEDV, right ventricular end-diastolic volume; RVESV, right ventricular end-systolic volume; *, statistically significant difference compared to baseline; #, statistically significant difference compared to 6 months postoperatively; †, statistically significant difference compared to 1 year postoperatively; ‡, statistically significant difference compared to 2 years postoperatively. | | | | | | | |

| **Supplementary Table S2. Evolution of Right Ventricular Diastolic Function and Myocardial Performance at Different Timepoints** | | | | | | |
| --- | --- | --- | --- | --- | --- | --- |
|  | **Postoperative Timepoints** | | | | | ***P* value** |
|  | **Postoperative**  **(n=200)** | **6 Months**  **(n=178)** | **1 year**  **(n=131)** | **2 years**  **(n=100)** | **3 years**  **(n=78)** |  |
| **Diastolic Function** |  |  |  |  |  |  |
| E [cm/s] | 54 (52, 55) | 54 (53, 54) | 54 (51, 54) | 54 (52, 55) | 54 (51, 55) | 0.34 |
| A [cm/s] | 38 (36, 40) | 38 (38, 40) | 38 (38, 40) | 38 (39, 40) | 38 (38, 40) | 0.83 |
| E/A Ratio | 1.4 (1.2, 1.6) | 1.4 (1.3, 1.5) | 1.4 (1.2, 1.5) | 1.4 (1.3, 1.5) | 1.4 (1.3, 1.5) | 0.42 |
| DT [ms] | 174 (165, 182) | 176 (170, 180) | 172 (172, 180) | 173 (172, 178) | 174 (168, 180) | 0.88 |
| E’ [cm/s] | 12 (11, 13) | 13 (12, 14) | 13 (12, 14) | 13 (12, 14) | 13 (12, 14) | 0.98 |
| A’ [cm/s] | 10 (9, 11) | 11 (10, 12) | 11 (10, 12) | 11 (10, 12) | 11 (10, 12) | 0.31 |
| E’/A' | 1.2 (1.1, 1.3) | 1.2 (1.1, 1.3) | 1.2 (1.1, 1.3) | 1.2 (1.1, 1.3) | 1.2 (1.1, 1.3) | 0.53 |
| E/E’ | 4.2 (4.4, 4.6) | 4.2 (4.1, 4.3) | 4.2 (4.1, 4.3) | 4.2 (4.1, 4.3) | 4.2 (4.1, 4.3) | 0.65 |
| **Myocardial Performance** |  |  |  |  |  |  |
| IVCT [ms] | 40 (30, 50) | 41 (32, 52) | 42 (33, 53) | 42 (33, 53) | 42 (33, 53) | 0.68 |
| IVRT [ms] | 80 (68, 88) | 79 (71, 89) | 81 (72, 92) | 81 (72, 92) | 82 (72, 91) | 0.72 |
| ET [ms] | 322 (310, 340) | 323 (312, 342) | 324 (311, 345) | 325 (312, 346) | 325 (313, 343) | 0.65 |
| MPI | 0.49 ± 0.13 | 0.50 ± 0.12 | 0.50 ± 0.12 | 0.53 ± 0.12 | 0.52 ± 0.11 | 0.77 |
| Note: E, tricuspid valve early diastolic filling peak velocity; A, tricuspid valve late diastolic filling peak velocity; DT, deceleration time; E’, tissue Doppler-derived tricuspid annular early diastolic myocardial velocity; A’, tissue Doppler-derived tricuspid annular late diastolic myocardial velocity; IVCT, isovolumic contraction time; IVRT, isovolumic relaxation time; ET, ejection time; MPI, myocardial performance index (i.e., Tei index). | | | | | | |

| **Supplementary Table S3. Three-Dimensional Right Ventricular Normal Reference Values in Heart Transplant Patients by Gender** | | | | |
| --- | --- | --- | --- | --- |
|  | **Overall Population**  **（N=500）** | **Males**  **（N=397）** | **Females**  **（N=103）** | ***P-value*** |
| RVEDV (mL) | 87 (51, 143) | 91 (54, 146) | 83 (48, 140) | **< 0.05** |
| RVESV (mL) | 42 (23, 75) | 45 (26, 72) | 39 (20, 78) | **< 0.05** |
| RVEF (%) | 52 (40, 62) | 50 (39, 60) | 53 (41, 64) | **< 0.05** |
| LEF (%) | 18 (6, 28) | 17 (6, 28) | 22 (6, 28) | **< 0.05** |
| REF (%) | 24 (9, 36) | 23 (7, 33) | 28 (11, 38) | **< 0.05** |
| AEF (%) | 23 (11, 34) | 22 (9, 32) | 27 (13, 34) | **< 0.05** |
| 3D-RVGLS (%) | −16 (−23, −8) | −15 (−21, −6) | −20 (−25, −10) | **< 0.05** |
| Note: Data are presented as median (interquartile range); 3D-RVGLS, three-dimensional right ventricular global longitudinal strain; AEF, anteroposterior ejection fraction; LEF, longitudinal ejection fraction; REF, radial ejection fraction; RVEDV, right ventricular end-diastolic volume; RVEF, right ventricular ejection fraction; RVESV, right ventricular end-systolic volume. | | | | |

| **Supplementary Table S4. Normal Reference Values for 3D Right Ventricular Structure and Function**  **in Adult Heart Transplant Patients by Age Group** | | | | | | | | | |
| --- | --- | --- | --- | --- | --- | --- | --- | --- | --- |
|  | **Males（N=397）** | | | |  | **Female（N=103）** | | | |
|  | **18-40 Years** | **41-65 Years** | **＞65 Years** | **ANOVA *P* value** |  | **18-40 Years** | **41-65 Years** | **＞65 Years** | **ANOVA *P-value*** |
|  | **（N=239）** | **（N=120）** | **（N=38）** |  |  | **（N=60）** | **（N=25）** | **（N=18）** |  |
| RVEDV (mL) | 92 (55, 147) | 90 (53, 145) | 85 (51, 143) | 0.354 |  | 83 (48, 140) | 84 (48, 140) | 81 (48, 140) | 0.656 |
| RVESV (mL) | 44 (23, 75) | 45 (25, 72) | 46 (24, 71) | 0.664 |  | 39 (20, 82) | 39 (21, 76) | 39 (18, 73) | 0.789 |
| RVEF (%) | 52 (41, 62) | 50 (39, 60)† | 45 (35, 55)†‡ | **< 0.05** |  | 53 (40, 63) | 54 (42, 66)† | 51 (39, 60)†‡ | **< 0.05** |
| LEF (%) | 18 (8, 30) | 16 (5, 27)† | 14 (5, 25)†‡ | **< 0.05** |  | 23 (7, 29) | 22 (6, 28)† | 18 (5, 24)†‡ | **< 0.05** |
| REF(%) | 24 (8, 34) | 22 (7, 32)† | 20 (6, 30)†‡ | **< 0.05** |  | 29 (12, 39) | 28 (11, 38)† | 24 (9, 35)†‡ | **< 0.05** |
| AEF (%) | 23 (10, 33) | 21 (9, 30)† | 19 (8, 28)†‡ | **< 0.05** |  | 28 (14, 35) | 27 (13, 34)† | 23 (11, 30)†‡ | **< 0.05** |
| RVGLS_3d_ (%) | −16 (−22, −7) | −14 (−20, −6)† | −12 (−18, −5)†‡ | **< 0.05** |  | −21 (−26, −11) | −20 (−25, −10)† | −16 (−19, 7)†‡ | **< 0.05** |
| Note: Data are presented as median (2.5th percentile, 97.5th percentile). RVGLS3d, three-dimensional right ventricular global longitudinal strain; AEF, anteroposterior ejection fraction; LEF, longitudinal ejection fraction; REF, radial ejection fraction; RVEDV, right ventricular end-diastolic volume; RVEF, right ventricular ejection fraction; RVESV, right ventricular end-systolic volume; †P < 0.05, compared to 18-40 years group; ‡P < 0.05, compared to 41-65 years group. | | | | | | | | | |

| **Supplementary Table S5. Intra- and Inter-Observer Reproducibility Analysis of 3D Right Ventricular Contraction Pattern Parameters** | | | | |
| --- | --- | --- | --- | --- |
|  | **Correlation Coefficient**  **(95% CI)** | **Bias** | **Limits of Agreement** | **Mean Absolute Deviation**  **(95% CI)** |
| **Intra-Observer** |  |  |  |  |
| RVEF | 0.95  (0.89 - 0.98) | -0.08 | -2.60 - 2.43 | -0.08  (-0.566 - 0.386) |
| LEF | 0.97  (0.92 - 0.99) | 0.22 | -1.32 - 0.80 | 0.22  (-0.065 - 0.518) |
| REF | 0.98  (0.93 - 0.99) | 0.07 | -1.15 - 1.31 | 0.07  (-0.152 - 0.304) |
| AEF | 0.92  (0.82 - 0.96) | -0.17 | -1.25 - 1.89 | -0.17  (-0.769 - 0.400) |
| **Inter-Observer** |  |  |  |  |
| RVEF | 0.93  (0.84 - 0.97) | 0.01 | -3.10 - 3.12 | 0.01  (-0.571 - 0.618) |
| LEF | 0.97  (0.92 - 0.99) | 0.42 | -1.12 - 1.00 | 0.42  (-0.116 - 0.708) |
| REF | 0.97  (0.93 - 0.99) | 0.24 | -1.02 - 1.37 | 0.24  (-0.001 - 0.404) |
| AEF | 0.90  (0.79 - 0.96) | -0.21 | -1.75 -1.41 | -0.31  (-0.968 - 0.241) |
| Note: AEF, anteroposterior ejection fraction; CI, confidence interval; LEF, longitudinal ejection fraction; REF, radial ejection fraction; RVEF, right ventricular ejection fraction; SD, standard deviation. | | | | |

| **Supplementary Table S6. Baseline Characteristics of Completers vs. Non-Completers at 3-Year Post-transplant** | | | |  |
| --- | --- | --- | --- | --- |
|  | **Completers** | **Non-completers** | ***P* value** | |
|  | **（N=78）** | **（N=122）** |  |  |
| **Demographics** |  |  |  | |
| Age [years] | 46.0 ± 11.5 | 47.8 ± 12.0 | 0.44 | |
| Gender [n(%)] |  |  |  | |
| Male | 64 (82) | 97 (80) | 0.65 | |
| Female | 14 (18) | 25 (20) |  | |
| **Etiology of Transplantation** |  |  |  | |
| Dilated Cardiomyopathy [n(%)] | 52 (67) | 70 (57) | 0.02 | |
| Hypertrophic Cardiomyopathy [n(%)] | 0 (0) | 2 (2) |  | |
| Ischemic Cardiomyopathy [n(%)] | 12 (15) | 33 (27) |  | |
| Valvular Cardiomyopathy [n(%)] | 13 (17) | 9 (7) |  | |
| Others [n(%)] | 1 (1) | 8 (7) |  | |
| **Comorbidities** |  |  |  | |
| Hypertension [n(%)] | 22 (28) | 42 (34) | 0.36 | |
| Hyperlipidemia [n(%)] | 45 (58) | 73 (60) | 0.76 | |
| Hyperglycemia [n(%)] | 34 (44) | 63 (52) | 0.27 | |
| Cerebrovascular Disease [n(%)] | 6 (8) | 16 (13) | 0.23 | |
| Renal Dysfunction [n(%)] | 25 (32) | 37 (30) | 0.79 | |
| **Transplantation Technique** |  |  |  | |
| Bicaval [n(%)] | 72 (92) | 100 (82) | 0.65 | |
| Biatrial [n(%)] | 6 (8) | 22 (18) |  | |

**SUPPLEMENTARY FIGURE 1 Study flowchart**

**SUPPLEMENTARY FIGURE 2 Comparison of three-dimensional right ventricular contraction patterns among transplant patients, cardiac surgery patients, and healthy controls at 1-year post-transplant**
